# A Population-Based, Nationwide Cross-Sectional Study on Influenza Vaccination Status among Cancer Survivors in Korea

**DOI:** 10.3390/ijerph120810133

**Published:** 2015-08-21

**Authors:** Myeung Guen Oh, Mi Ah Han, Na-Ra Yun, Jong Park, So Yeon Ryu, Dong-Min Kim, Seong-Woo Choi

**Affiliations:** 1 Department of Internal Medicine, Jeongup Asan Hospital, Jeongup 56153, Korea; E-Mail: medi2006@naver.com; 2 Department of Preventive Medicine, College of Medicine, Chosun University, Gwangju 61452, Korea; E-Mails: Jpark@chosun.ac.kr (J.P.); canrsy@chosun.ac.kr (S.Y.R.); jcsw74@chosun.ac.kr (S.-W.C.); 3 Department of Internal Medicine, Chosun University Hospital, Gwangju 61453, Korea; E-Mails: shine@chosun.ac.kr (N.-R.Y.); drongkim@chosun.ac.kr (D.-M.K.)

**Keywords:** influenza, human, influenza vaccines, neoplasms, survivors

## Abstract

Cancer survivors are at increased risk of developing influenza-related complications. The purpose of this study was to investigate the vaccination coverage among cancer survivors in Korea using the Korea National Health and Nutrition Examination Survey (KNHANES). Adult cancer survivors were selected from fourth (2007–2009) and fifth (2010–2012) KNHANES (*n* = 1156) datasets. General characteristics, cancer-related data, and influenza vaccination status were collected using self-report questionnaires. Chi-square tests and multiple logistic regression analyses were performed to investigate the association between influenza vaccination coverage and associated factors. Overall, 51% of survivors were vaccinated. Vaccine prevalence exceeded 75% in those more than 65 years but was only 26% in survivors aged 19–44. Increasing age, low frequency of alcohol consumption, having poor self-rated health, and having a shorter duration since cancer diagnosis were significant predictors of vaccination status among cancer survivors under 65 years of age. Influenza vaccine coverage remains much lower than recommended among cancer survivors, particularly in the younger age groups. Further study is needed to determine the factors that contribute to the lack of vaccination in cancer survivors, despite their increased risk for influenza.

## 1. Introduction

Influenza is a contagious viral respiratory illness. Despite increasing public awareness and an accessible, effective vaccine, influenza remains a significant cause of morbidity and mortality [1]. Worldwide, annual epidemics result in about three to five million cases of severe illness and about 250,000 to 500,000 influenza-related deaths [2]. Vaccination is the single most effective tool to prevent influenza and its associated complications and is recommended in older individuals and those with chronic medical conditions such as cancer [3,4,5].

In Korea, the five-year relative survival rate for cancer has steadily improved from 41.2% in 1993 to 1995, 53.7% in 2001 to 2005, and 64.1% in 2006 to 2010. These improvements in survival rate are due to early cancer detection by screening, along with advances in cancer treatment [6]. Cancer survivors, including those who are currently not undergoing treatment, are considered a high-risk group for influenza infection and its complications, including death and hospitalization [4,7,8]. Compared with the general population, patients with cancer have an increased risk of complications that lead to additional morbidity and mortality and can delay or interrupt cancer treatment [8]. Current recommendations advise people with cancer or a history of cancer to receive the influenza vaccination [7]. Death from influenza-related infections occurs in an estimated 9% of cancer patients [9], and a high vaccination coverage in the broader population likely increases the benefit derived from the direct influenza vaccination [1]. In previous studies in the United States, 57.8% of cancer survivors reported receiving an influenza vaccination during the previous year [10] and about 75% of cancer survivors in the United Kingdom had received an influenza vaccination [11].

In Korea, annual vaccination against influenza is universally recommended for all adults. In particular, vaccination is recommended for elderly individuals and individuals with chronic diseases because they have a high risk for influenza-related complications [12]. Influenza coverage for Korean individuals ≥65 years has improved over recent years and is among the highest in the world [13]. However, the vaccination coverage for those <65 years is lower than 30%, even in individuals with associated chronic conditions [14]. In a previous Korean study, only 34.1% of outpatients in cancer centers received the 2009 H1N1 vaccination [8], and data on seasonal influenza vaccination coverage among cancer survivors is limited. This study therefore sought to investigate factors associated with vaccination coverage among cancer survivors in Korea using national representative data. These data may help determine the influenza vaccination rate among cancer survivors and identify those for whom efforts to increase influenza vaccination should be intensified.

## 2. Materials and Methods

### 2.1. Data Source

We used data from the Korea National Health and Nutrition Examination Survey (KNHANES), a population-based cross-sectional survey designed to assess health-related behavior, health conditions, and nutritional state of Koreans. The survey was conducted as a triennial survey for the first three cycles implemented in 1998, 2001, and 2005, respectively, and was converted to an annual survey beginning with the fourth cycle in 2007 [15]. We used data from the fourth (2007–2009) and fifth (2010–2012) KNHANES. From 2007 the survey became a continuous program covering about 10,000 individuals each year, except for the year 2007, when the number of participants was half of that of other years as the 2007 survey was conducted during a half-year (from July through December) [16].

A total of 18,406 adults and 19,599 adults participated in the fourth and fifth KNHANES, respectively, for overall response rates of 78.4% (24,871 of 31,705) and 80.8% (25,534 of 31,596), respectively. Of these, 2628 adults (1095 in the fourth KNHANES and 1533 adults in the fifth KNHANES) had missing data and 250 adults responded “Don’t know” regarding vaccination status. Finally, 35,127 adults (age ≥ 19) were analyzed in this study. KNHANES was approved by the Korea Centers for Disease Control and Prevention Institutional Review Board and all participants signed a written informed consent form.

### 2.2. Cancer Survivors and Cancer-Free Comparison Group

Cancer survivors were defined as “if they had been ever diagnosed with cancer by a doctor”. Data regarding cancer site and age at diagnosis were also collected. Time since diagnosis was calculated by subtracting the patient’s age at diagnosis from their current age. If survivors had had multiple cancers, the age at which they had been diagnosed the first time was used. Participants who had no history of cancer were classified into the cancer-free comparison group.

### 2.3. Influenza Vaccination

Influenza vaccination was defined as a positive response to the following question: “During the past 12 months, did you receive an influenza vaccine (a flu shot)?” Respondents who replied “don’t know” or “refused” were excluded from the analysis.

### 2.4. Covariates

The following variables were included in the analyses: sex, age, marital status (without spouse, with spouse), education (≤elementary school, middle school, ≥high school), household income (low, middle-low, middle-high, high), health insurance (National Health Insurance, Medical Aid Program), smoking status (never, former, current), drinking frequency (none, ≤1/month, ≥2/month), physical activity (no, yes), medical checkup (no, yes), non-cancer, co-morbid condition (subjects with hypertension, diabetes mellitus, heart diseases, stroke, asthma, pulmonary tuberculosis, chronic obstructive pulmonary disease, hepatitis B, C, liver cirrhosis and renal failure), self-rated health (good, fair, poor) and survey year.

### 2.5. Statistical Analysis

We first described and compared the distribution of the study variables in cancer survivors (n = 1156) and subjects without a history of cancer (*n* = 33,971). Chi-square tests were used to determine whether the demographic and socioeconomic characteristics differed among survivors and non-cancer comparison group. Second, influenza vaccination coverage among cancer survivors and non-cancer comparison groups were calculated and compared by chi-square tests. Finally, we used multiple logistic regression analyses to investigate factors associated with influenza vaccination in cancer survivors and non-cancer comparison groups. Because the vaccine is administered free of charge for subjects ≥65 years in Korea, we stratified our subjects by age group (<65 years *vs.* ≥65 years) to analyze vaccination coverage and associated factors. All statistics with the exception of crude numbers were weighted to provide national estimates based on the sampling method. All statistical analyses were performed using SAS 9.2 (SAS Institute, Cary, NC, USA).

## 3. Results

### 3.1. Characteristics of Cancer Survivors

The overall study sample comprised 1156 cancer survivors and 33,971 individuals who had no previous history of cancer. Women accounted for 64.2% of the cancer survivor group and 50.3% of the non-cancer comparison group. The median age was 58.4 years (interquartile range 48.3–68.5 years) for cancer survivors, and 42.5 years (interquartile range 31.3–54.7 years) for the non-cancer comparison group (Table 1).

**Table 1:** Characteristics of study subjects by cancer history in Korea National Health and Nutrition Examination Survey.

Characteristics	Cancer Survivors (*n* = 1156)	No History of Cancer (*n* = 33,971)	*p*-Value
Sex			<0.001
Men	412(35.8)	14,464(49.7)	
Women	744(64.2)	19,507(50.3)	
Age (years)			<0.001
19–44	123(14.9)	14,382(53.1)	
45–64	510(48.4)	11,940(3.7)	
≥65	523(36.7)	7649(13.2)	
Medians (interquartile range)	58.4(48.3–68.5)	42.5(31.3–54.7)	
Marital status			<0.001
Without spouse	258(23.1)	9289(32.1)	
With spouse	897(76.9)	24,576(67.9)	
Education			<0.001
≤Elementary school	500(38.1)	9134(19.0)	
Middle school	175(15.5)	3699(10.1)	
≥High school	480(46.4)	21,097(70.9)	
Household income			<0.001
Low	346(27.1)	6720(15.9)	
Middle-low	286(26.6)	8476(26.3)	
Middle-high	242(21.4)	9044(29.2)	
High	259(24.9)	9129(28.7)	
Health insurance			<0.001
National Health Insurance	1080(93.1)	32,636(96.5)	
Medical Aid Program or none	74(6.9)	1325(3.5)	
Smoking status			<0.001
Never	734(65.6)	19,900(53.2)	
Former	315(25.2)	6643(19.7)	
Current	103(9.1)	7374(27.2)	
Drinking frequency			<0.001
None	609(49.9)	9669(23.1)	
≤1/month	293(26.4)	9677(28.6)	
≥2/month	248(23.7)	14,483(48.3)	
Physical activity			0.595
No	912(78.8)	26,638(78.0)	
Yes	244(21.2)	7333(22.0)	
Medical checkup			<0.001
No	436(40.1)	15,039(47.8)	
Yes	720(59.9)	18,932(52.2)	
Non-cancer, co-morbid conditions ^a^			<0.001
No	638(60.7)	24,490(78.1)	
Yes	518(39.3)	9481(21.9)	
Self-rated health			<0.001
Good	267(22.9)	12,504(37.7)	
Fair	406(36.6)	14,176(43.9)	
Poor	483(40.5)	7286(18.4)	
Year of survey			0.072
2007	78(6.9)	2904(9.0)	
2008	196(16.3)	6605(18.2)	
2009	230(17.2)	7242(18.3)	
2010	214(19.4)	6037(18.3)	
2011	223(20.7)	5797(18.3)	
2012	215(19.6)	5386(18.0)	

Data are expressed as n (weighted %). ^a^ Non-cancer co-morbid conditions included hypertension, diabetes mellitus, heart diseases, stroke, asthma, pulmonary tuberculosis, chronic obstructive pulmonary disease, hepatitis B, C, liver cirrhosis and renal failure.

The cancer survivor and non-cancer comparison groups had very different demographic and health-related characteristics. Cancer survivors were older and more likely to be female, have less education, earn a lower monthly income, and have a poor self-reported health status. In addition, cancer survivors were less likely to be current smokers or drinkers than the non-cancer comparison group (Table 1). A total of 20% of cancer survivors were diagnosed with cancer within the past year, and 51% had been diagnosed more than 5 years prior to the study. By age group, 21.8% and 46.6% of survivors <65 years were diagnosed within 1 year and >5 years, respectively. Among older patients, 17.1% and 60.0% of survivors ≥65 years old were diagnosed within 1 year and >5 years, respectively. The most common cancer site was the stomach (Table 2).

**Table 2:** Clinical characteristics of cancer survivors in Korea National Health and Nutrition Examination Survey.

Characteristics	*N* (Weighted %)
Age at cancer diagnosis (years)	
19–44	292(32.1)
45–64	638(52.1)
≥65	225(15.8)
Medians (interquartile range)	50.2(41.6–60.9)
Time since diagnosis (years)	
≤1	219(20.1)
<5	311(28.5)
≥5	625(51.5)
Medians (interquartile range)	4.2(1.5–9.7)
Cancer site	
Stomach	226(17.7)
Liver	35(3.7)
Colon	108(9.5)
Breast	152(13.2)
Cervix	144(12.7)
Lung	33(2.1)
Thyroid	51(3.8)
Other	407(37.1)

### 3.2. Vaccination Coverage in Cancer Survivors Compared with Non-Cancer Comparison Group

Overall vaccination coverage among cancer survivors was 51.2%. With regard to age, vaccination coverage of the cancer survivor group (35.8%) was significantly higher compared to that of the non-cancer comparison group (22.9%, *p* < 0.001) in subjects <65 years. Vaccination coverage in subjects ≥65 years was similar between cancer survivor group (77.7%) and the non-cancer comparison group (75.8%, *p* = 0.486) (Table 3).

**Table 3:** Influenza vaccination coverage by cancer history in Korea National Health and Nutrition Examination Survey.

Characteristics	Age < 65						Age ≥ 65				
	Cancer Survivors (*n* = 633)			No Cancer (*n* = 26,322)			Cancer Survivors (*n* = 523)			No Cancer (*n* = 7649)	
	Vaccinated % (SE)	*p*-Value		Vaccinated % (SE)	*p*-Value		Vaccinated % (SE)	*p*-Value		Vaccinated % (SE)	*p*-Value
Total	35.8(2.4)			22.9(0.4)			77.7(2.5)			75.8(0.7)	
Sex		0.490			<0.001			0.526			0.002
Men	38.3(4.7)			20.0(0.5)			79.3(3.5)			73.6(1.0)	
Women	34.8(2.6)			26.0(0.5)			76.3(3.3)			77.3(0.8)	
Age (years)		0.028			<0.001						
19–44	26.1(4.8)			19.5(0.5)							
45–64	38.8(2.6)			28.3(0.6)							
≥65							77.7(2.5)			75.8(0.7)	
Marital status		0.548			<0.001			0.881			0.826
Without spouse	38.9(5.7)			17.1(0.6)			78.2(4.0)			75.6(1.0)	
With spouse	35.2(2.6)			25.6(0.4)			77.5(3.1)			75.9(0.8)	
Education		0.026			<0.001			0.719			0.863
≤Elementary school	46.0(4.8)			35.6(1.1)			78.4(3.0)			76.0(0.8)	
Middle school	36.6(4.8)			29.2(1.1)			79.8(6.1)			75.0(1.8)	
≥High school	31.6(3.2)			20.4(0.4)			74.3(5.4)			75.6(1.6)	
Household income		0.394			0.842			0.940			0.811
Low	36.8(5.9)			23.5(1.1)			76.8(3.2)			76.6(0.9)	
Middle-low	42.1(4.8)			23.2(0.7)			77.5(5.7)			75.4(1.3)	
Middle-high	35.2(4.6)			22.6(0.6)			74.2(6.3)			75.2(1.9)	
High	31.7(4.1)			23.0(0.6)			80.2(6.9)			75.4(2.2)	
Health insurance		0.945			0.033			0.923			0.111
National Health Insurance	35.8(2.4)			22.8(0.4)			77.5(2.7)			76.1(0.7)	
Medical Aid Program or none	36.5(10.2)			27.2(2.1)			78.3(7.0)			72.3(2.4)	
Smoking status		0.252			<0.001			0.676			0.003
Never	35.9(2.7)			25.5(0.5)			77.6(3.2)			76.7(0.8)	
Former	40.6(5.1)			24.3(0.8)			78.6(4.0)			76.2(1.2)	
Current	25.6(7.4)			17.3(0.6)			70.9(8.4)			70.9(1.7)	
Drinking frequency		0.038			<0.001			0.558			0.816
None	39.0(3.6)			27.9(0.8)			75.8(3.1)			75.9(0.9)	
≤1/month	39.7(4.1)			24.2(0.6)			82.1(4.7)			76.2(1.4)	
≥2/month	26.4(4.2)			20.4(0.4)			79.1(5.7)			75.2(1.1)	
Physical activity		0.085			0.832			0.833			0.918
No	33.6(2.5)			22.9(0.4)			77.9(2.9)			75.8(0.7)	
Yes	42.9(5.1)			23.1(0.7)			76.6(5.3)			75.7(1.6)	
Medical checkup		0.160			<0.001			0.086			<0.001
No	31.4(3.8)			17.5(0.5)			73.2(4.1)			68.4(1.0)	
Yes	38.5(3.0)			28.0(0.5)			81.1(2.8)			81.4(0.8)	
Non-cancer, co-morbid conditions^a^		0.095			<0.001			0.155			<0.001
No	33.5(2.8)			21.1(0.4)			73.4(4.5)			72.6(1.0)	
Yes	42.1(4.4)			32.8(0.9)			80.4(2.8)			77.7(0.8)	
Self-rated health		0.061			<0.001			0.998			<0.001
Good	27.5(4.1)			20.9(0.5)			77.4(5.5)			71.6(1.2)	
Fair	36.0(3.7)			23.3(0.5)			77.8(4.7)			76.8(1.0)	
Poor	41.6(4.0)			27.0(0.8)			77.7(3.2)			78.1(1.0)	
Year of survey		0.730			<0.001			0.010			0.002
2007	31.9(9.1)			22.8(1.0)			75.4(6.9)			69.9(2.3)	
2008	30.0(4.9)			22.3(0.8)			89.3(4.0)			72.6(1.6)	
2009	42.1(5.0)			20.0(0.7)			70.2(5.2)			74.6(1.7)	
2010	36.0(5.5)			21.8(0.8)			63.0(7.7)			78.0(1.5)	
2011	38.1(5.6)			24.7(1.0)			82.2(4.7)			79.4(1.4)	
2012	33.8(5.5)			26.1(0.9)			83.3(5.5)			76.9(1.7)	
Time since last diagnosis (years)		0.173						0.338			
≤1	42.5(5.2)						74.5(5.6)				
<5	30.8(3.8)						83.5(4.1)				
≥5	36.1(3.3)						76.3(3.4)				
Cancer site		0.264						0.936			
Stomach	42.3(6.4)						79.4(5.0)				
Liver	27.8(11.3)						85.4(9.7)				
Colon	40.3(8.9)						75.2(6.1)				
Breast	32.5(5.5)						78.0(7.7)				
Cervix	27.0(4.6)						72.0(7.0)				
Lung	63.9(12.6)						86.4(7.1)				
Thyroid	43.8(9.3)						76.0(12)				
Other	35.7(3.9)						77.9(4.1)				

SE, standard error.

In multiple logistic regression analysis with subjects aged <65 years, cancer survivors were more likely to receive an influenza vaccination than the non-cancer comparison group (adjusted OR = 1.33, 95% CI = 1.07–1.66) after adjusting for demographic and health-related characteristics. In subjects ≥65 years, the likelihood of receiving vaccination was similar among cancer survivor and non-cancer comparison groups (adjusted OR = 1.06, 95% CI = 0.78–1.44).

### 3.3. Factors Associated with Influenza Vaccination in Cancer Survivors and Non-Cancer Comparison Group

A linear trend toward increased vaccination with older age was observed in both cancer survivors and adults without cancer. Low frequency of alcohol consumption and poor self-rated health were also associated with increased vaccination in both groups. With regard to cancer-related factors, shorter duration since diagnosis was associated with increased likelihood of receiving the influenza vaccine in cancer survivors <65 years. Vaccination coverage was higher in cancer survivors diagnosed within 1 year compared to subjects diagnosed more than 5 years ago (aOR = 1.74, 95% CI = 1.01–3.00).

In subjects with no history of cancer, prevalence of vaccination coverage was higher in those that were women, married, less educated, and non-smokers, as well as those who received regular medical checkups, had a non-cancer co-morbidity, and were surveyed in the more recent KNHANES. However, none of these associations was observed among adult cancer survivors (Table 4).

**Table 4:** Associated factors with influenza vaccination of Korea National Health and Nutrition Examination Survey.

Characteristics	Age < 65			Age ≥ 65	
	Cancer Survivors	No Cancer		Cancer Survivors	No Cancer
Sex					
Men	1.00	1.00		1.00	1.00
Women	0.48(0.22–1.04)	1.26(1.14–1.39) ^a^		0.49(0.15–1.60)	1.48(1.19–1.83) ^a^
Age (years)	1.06(1.03–1.09) ^a^	1.01(1.01–1.01) ^a^		1.04(0.98–1.11)	1.05(1.03–1.06) ^a^
Marital status					
Without spouse	1.00	1.00		1.00	1.00
With spouse	0.58(0.32–1.06)	1.27(1.15–1.40) ^a^		0.99(0.49–2.00)	1.19(0.99–1.41)
Education					
≤Elementary school	1.28(0.73–2.24)	1.52(1.35–1.72) ^a^		1.87(0.88–3.98)	0.94(0.77–1.16)
Middle school	0.77(0.45–1.32)	1.29(1.15–1.46) ^a^		1.60(0.62–4.14)	0.95(0.73–1.23)
≥High school	1.00	1.00		1.00	1.00
Household income					
Low	0.85(0.40–1.81)	0.90(0.78–1.03)		1.05(0.34–3.25)	1.08(0.84–1.40)
Middle-low	1.49(0.78–2.84)	0.98(0.89–1.09)		1.12(0.35–3.63)	1.04(0.79–1.37)
Middle-high	1.28(0.70–2.35)	0.99(0.91–1.09)		0.93(0.26–3.31)	1.01(0.74–1.38)
High	1.00	1.00		1.00	1.00
Health insurance					
National Health Insurance	0.83(0.29–2.37)	0.64(0.50–0.81) ^a^		1.13(0.42–3.04)	1.34(1.01–1.77) ^a^
Medical Aid Program or none	1.00	1.00		1.00	1.00
Smoking status					
Never	2.14(0.90–5.11)	1.28(1.13–1.45) ^a^		2.72(0.66–11.16)	1.07(0.84–1.36)
Former	1.11(0.46–2.64)	1.32(1.18–1.49) ^a^		1.50(0.58–3.88)	1.22(0.99–1.51)
Current	1.00	1.00		1.00	1.00
Drinking frequency					
None	1.89(1.05–3.40)^a^	1.14(1.03–1.26) ^a^		0.85(0.40–1.83)	0.91(0.76–1.08)
≤1/month	2.58(1.38–4.83)^a^	1.09(1.01–1.19) ^a^		1.30(0.51–3.30)	0.96(0.78–1.17)
≥2/month	1.00	1.00		1.00	1.00
Physical activity					
No	1.00	1.00		1.00	1.00
Yes	1.54(0.95–2.51)	1.06(0.97–1.15)		1.02(0.49–2.12)	1.07(0.88–1.29)
Medical checkup					
No	1.00	1.00		1.00	1.00
Yes	1.39(0.85–2.28)	1.61(1.49–1.75) ^a^		1.53(0.86–2.73)	2.16(1.87–2.48) ^a^
Non-cancer, co-morbid conditions^a^					
No	1.00	1.00		1.00	1.00
Yes	1.01(0.62–1.66)	1.40(1.28–1.54) ^a^		1.57(0.88–2.80)	1.24(1.08–1.43) ^a^
Self-rated health					
Good	1.00	1.00		1.00	1.00
Fair	1.96(1.11–3.46)^a^	1.05(0.97–1.14)		0.94(0.42–2.09)	1.27(1.07–1.50) ^a^
Poor	2.26(1.31–3.91)^a^	1.10(0.99–1.21)		0.88(0.41–1.89)	1.39(1.18–1.64) ^a^
Year of survey					
2007	1.00	1.00		1.00	1.00
2008	0.70(0.26–1.90)	0.98(0.84–1.14)		2.26(0.68–7.50)	1.09(0.82–1.44)
2009	1.19(0.47–3.04)	0.85(0.73–0.98)		0.68(0.24–1.89)	1.13(0.85–1.50)
2010	1.13(0.42–3.03)	0.95(0.81–1.10)		0.42(0.14–1.25)	1.28(0.96–1.70)
2011	1.26(0.49–3.26)	1.13(0.96–1.33)		1.35(0.43–4.31)	1.39(1.05–1.85) ^a^
2012	0.89(0.32–2.49)	1.22(1.04–1.42) ^a^		1.47(0.41–5.28)	1.16(0.87–1.54)
Time since last diagnosis (years)					
≤1	1.74(1.01–3.00) ^a^			0.72(0.34–1.50)	
<5	0.90(0.53–1.52)			1.18(0.60–2.33)	
≥5	1.00			1.00	
Cancer site					
Stomach	1.00			1.00	
Liver	1.35(0.72–2.52)			1.03(0.48–2.20)	
Colon	0.57(0.16–2.07)			3.01(0.68–13.21)	
Breast	1.42(0.56–3.56)			0.75(0.29–1.91)	
Cervix	0.87(0.43–1.77)			1.31(0.44–3.84)	
Lung	0.78(0.39–1.59)			0.72(0.25–2.03)	
Thyroid	3.49(0.59–20.79)			2.35(0.57–9.68)	
Other	2.01(0.83–4.89)			0.71(0.16–3.18)	

Data are expressed as adjusted odds ratio (95% confidence interval). ^a^ statistically significant at the 95 confidence level.

## 4. Discussion

This study investigated the factors relating to influenza vaccine coverage among Korean cancer survivors. A total of 36% and 78% of cancer survivors <65 and ≥65 years, respectively, had been vaccinated within the past 12 months. Age, frequency of alcohol consumption, self-rated health, and time since diagnosis were associated with influenza vaccination in cancer survivors.

Overall vaccination coverage among cancer survivors was 51%, which was higher than the subjects without a history of cancer. Influenza vaccination coverage increased significantly with age, from 26.1% of those 19–49 years to 77.7% among respondents ≥65 years. These coverage rates are far lower than what is recommended in a population at an increased risk for influenza-related complications. Although the goal of the Health Plan 2020 (Korea National Health Promotion Plan) is to increase the vaccination coverage up to 82.5% in elderly individuals age 65 and over, optimal vaccination coverage for cancer survivors in Korea is unspecified [17]. According to the 2009 Behavioral Risk Factor Surveillance System survey, the overall vaccination coverage of cancer survivors was 64% in US: from 31% of those aged 18–34 to 74% among respondents aged 65 and older [4], indicating that the vaccination coverage of Korean cancer survivors is lower than that of US survivors especially in younger age groups.

Vaccination coverage in younger cancer survivors was significantly lower than that of older survivors, which is consistent with previous studies. The higher vaccination coverage in those ≥65 years may be because vaccination is universally recommended for these individuals in Korea and many other countries [18,19,20]. Older subjects (≥65 years) are vaccinated free of charge in Korea, but younger age groups are not similarly supported. Hence, older subjects may be more likely to receive vaccinations due to their age rather than cancer prevalence *per se*. The lower vaccination coverage in younger survivors might be due to less social support, differences in health status, and provider bias.

Previous studies show that adult vaccinations, including influenza, are influenced by provider-based recommendation, as heath professional recommendation is the most important and effective factor encouraging vaccination [14]. However, a previous Korean study indicates some oncologists do not know that cancer patients in all age groups should receive the influenza vaccine [8]. Further studies should investigate the relationship between vaccination status and provider awareness.

Poor self-rated health was significantly associated with higher vaccination coverage in cancer survivors. Although, KNHANES did not collect detailed clinical information such as cancer stage, current cancer status, or presence of symptoms, self-rated health maybe a reflection of current general health due to cancer. In previous studies, those who receive the vaccination have poorer self-rated health than their unvaccinated counterparts [21].

Vaccination coverage was higher in individuals who had been diagnosed with cancer more recently. Patients who are diagnosed within a year are likely to be under active treatment and are therefore at a high risk of immunosuppression. In a recent review, the majority of cancer patients who have not received treatment for more than 30 days mount immunologically favorable reactions to the influenza vaccination. Although patients actively receiving treatment typically have suboptimal responses, few studies show a complete lack of response. Although the ideal time to administer the vaccine during a treatment cycle is unclear, most studies recommend routine administration of the influenza vaccine [1]. Also, recent cancer diagnosis may have increased awareness among cancer survivors of their increased risk for influenza. Moreover, patients with cancer undergo multiple follow up visits during their cancer treatment. Oncologists may be more likely to vaccinate these high-risk patients, which may be partially responsible for the increased prevalence of vaccination in patients with cancer. Cancer survivors with longer duration since their initial diagnosis may be in remission and thus may have fewer hospital visits (and fewer opportunities for vaccination) than those recently diagnosed. Unfortunately, cancer treatment status and health professional-related factors were not investigated in KNHANES. The time since diagnosis is also the most important predictor of all other preventive health behaviors such as smoking, alcohol drinking, and physical activity in cancer survivors [22,23,24]. Therefore, time since diagnosis should be considered in the management of health behaviors among cancer survivors.

This study revealed different relationships between health insurance and influenza vaccination coverage according to age group. In the younger group, vaccination was significantly higher among Medical Aid Program (MAP) beneficiaries, while National Health Insurance (NHI) beneficiaries tended to be vaccinated more among the older group. The influenza vaccination was provided free of charge at public health centers for subjects known to be financially vulnerable, and nursing home residents received it according to local government policy and financial conditions [25,26]. The health insurance of these vulnerable people was generally covered by MAP. Overall, the younger subjects covered by MAP were more likely to be vaccinated compassred to NHI-covered subjects. In Korea, citizens older than 65 years can be vaccinated for free regardless of their health insurance type. In this study, NHI beneficiaries older than 65 were more likely to be vaccinated compared to MAP beneficiaries. The NHI beneficiaries also had higher levels of education and income, as previously described [27]. Those with a higher educational and/or income level are more likely to receive preventive health service including vaccination, possibly because they are more knowledgeable about health-related services and the effectiveness of preventive medicines [26,28]. Hence, NHI beneficiaries might have higher vaccination rates due to knowledge or awareness about its benefits. The findings of this study imply that public health policies should focus separately on the target groups according to age and health insurance type to improve influenza vaccination coverage.

There are some limitations associated with the use of KNHANES data. First, the survey did not include institutionalized or non-civilian individuals, so cancer survivors who were hospitalized, residing in a long-term care facility, or living in hospice care were not included. Male patients and older cancer survivors were more likely to be hospitalized [29]. Because long-term care facilities typically emphasize preventive care for infectious illnesses such as influenza, our sample is likely an underestimate of actual vaccine prevalence in the population [4]. Second, the use of self-reported data on vaccination could introduce possible bias. However, self-report of influenza vaccination status is a highly sensitive [30,31] and moderately specific measure [30]. Third, KNHANES did not collect the information about cancer treatment status or method. Several previous studies show that use of the influenza vaccination varies based on treatment status (untreated and actively treated patients) and type (chemotherapy or radiation therapy) [1], suggesting that these factors may affect vaccination status of cancer survivors and physician recommendations for vaccination.

We reported the representative rate of influenza vaccination among cancer survivors. Influenza vaccination coverage remains much lower than desired among cancer survivors; vaccine prevalence only exceeded 75% in those 65 years or older, and just 26% of younger survivors (19–44 years) had been vaccinated within the previous 12 months. Our study identified cancer survivors for whom influenza vaccination targeting efforts could be intensified. Younger cancer survivors and survivors with high frequency of alcohol consumption, good self-rated health, and longer time since diagnosis should be encouraged to get vaccinated. These findings also indicate a need for further research to understand why these individuals did not receive the vaccine despite their increased risk for influenza. Future studies should assess the effect of awareness and oncologist or physician recommendation on vaccination among cancer survivors.
